# Personalised insulin calculator enables safe and effective correction of hyperglycaemia prior to FDG PET/CT

**DOI:** 10.1186/s13550-019-0480-2

**Published:** 2019-02-08

**Authors:** David A. Pattison, Lisa L. MacFarlane, Jason Callahan, Emma L. Kane, Timothy Akhurst, Rodney J. Hicks

**Affiliations:** 1 Centre for Cancer Imaging, Peter MacCallum Cancer Centre, Melbourne, Australia; 2 Department of Nuclear Medicine & Specialised PET Services, Royal Brisbane & Women’s Hospital, Butterfield Street, Brisbane, Australia; 3 School of Medicine, University of Queensland, Brisbane, Australia; 4 School of Radiology, Health Education North East, Liverpool, UK; 5 The Sir Peter MacCallum Department of Oncology, University of Melbourne, Melbourne, Australia

**Keywords:** Insulin, Hyperglycaemia, FDG PET/CT, Diabetes mellitus

## Abstract

**Background:**

Hyperglycaemia can influence ^18^F-fluorodeoxyglucose (FDG) uptake due to competition for glucose transport and phosphorylation by hexokinase. Major international nuclear medicine societies recommend blood glucose level (BGL) < 11.1 mmol/L (200 mg/dL) prior to performing FDG positron emission tomography/computed tomography (PET/CT). However, there is no consensus approach and complications of previously proposed insulin guidelines included significant hypoglycaemia, inconvenience and skeletal muscle uptake. This study aims to establish the safety and efficacy of a personalised insulin calculator protocol to estimate the dose of intravenous insulin injection for correction of hyperglycaemia prior to FDG PET/CT.

**Results:**

This is a retrospective audit of all patients treated with insulin for hyperglycaemia (BGL > 10 mmol/L) prior to FDG PET/CT at the Peter MacCallum Cancer Centre over a 2-year period. Cohort 1 comprised a 12-month period (April 1, 2014–March 31, 2015) using the department’s established empiric-dose insulin protocol, and Cohort 2 the 12 months (April 1, 2015–March 31, 2016) following introduction of a personalised insulin calculator protocol. Variables including body mass index, insulin-dose calculated and/or administered, BGL at baseline and nadir, and time to FDG injection were analysed. There were 115 and 136 patients treated with insulin in Cohorts 1 and 2 respectively, with similar baseline variables including mean BGL (14.5 vs 14.4 mmol/L) and range (10.5–22.7 vs 10.4–24.3 mmol/L). Use of the new personalised insulin calculator resulted in significantly fewer hypoglycaemic events (0.7% vs 5.2%; *P* < 0.03), shorter median time from insulin to FDG injections (108 min vs 136 min; *P* < 0.001) and greater individualised range in insulin prescription (3–32 IU vs 4–20 IU). The majority of patients (88.3%) receiving the personalised insulin calculator prescribed dose achieved BGL < 10.0 mmol/L. All scans obtained were of diagnostic quality.

**Conclusions:**

The use of our personalised insulin calculator protocol effectively lowered BGL to the target range, resulted in significantly fewer hypoglycaemic events and reduced median time between insulin and FDG injection compared to a pre-existing empiric protocol whilst achieving diagnostic scans.

**Electronic supplementary material:**

The online version of this article (10.1186/s13550-019-0480-2) contains supplementary material, which is available to authorized users.

## Introduction

Imaging of glucose analogue ^18^F-fluorodeoxyglucose (FDG) metabolism using positron emission tomography/computed tomography (PET/CT) has an important role in the diagnosis, staging and response assessment of many common malignancies, in addition to a growing number of non-oncologic indications. There is also an alarming increasing global incidence of diabetes mellitus [1]. Hyperglycaemia is a common problem in patients with cancer, as a side-effect of numerous cancer therapies (including glucocorticoids and MTOR inhibitors [2]) and since there is an increased risk of cancer in patients with diabetes [3]. Hyperglycaemia may also potentially be exacerbated in patients presenting for FDG PET/CT scan after withholding metformin to reduce bowel uptake [4].

There is concern that hyperglycaemia can influence FDG uptake due to competition for glucose transport and phosphorylation by hexokinase. Numerous studies have demonstrated reduction in maximum standardised uptake value (SUVmax) and/or sensitivity for detection of malignancies in the pancreas [5, 6], lung [7], and head and neck [8] associated with hyperglycaemia. However, a similar SUVmax was observed in lung malignancies in a cohort of patients with well-controlled diabetes with euglycaemia and non-diabetic controls, suggesting the observation is due to hyperglycaemia rather than diabetes per se [9]. Rabkin et al. [10] also demonstrated that hyperglycaemia resulted in reduced detection of a variety of malignancies but did not influence the detection of infection or inflammation. Hyperglycaemia also compromises the consistency of SUV measurements, which, in turn, compromises the utility of FDG PET/CT in clinical trials.

Consequently, the consensus clinical guidelines of major societies recommend rescheduling patients with hyperglycaemia above specified thresholds (Society for Nuclear Medicine [SNM] < 150–200 mg/dL [11] [8.3–11.1 mmol/L], National Cancer Institute [NCI] < 200 mg/dL [12] [11.1 mmol/L]; European Association for Nuclear Medicine [EANM] < 11 mmol/L [about 200 mg/dL] for clinical studies or < 7.0–8.3 mmol/L [126–150 mg/dL] for clinical trials [13]; International Atomic Energy Agency [IAEA] < 200 mg/dL [14] [11.1 mmol/L]), or consideration of insulin administration for correction of hyperglycaemia. However, these guidelines provide limited guidance with respect to insulin dose and how long a delay should be instituted after insulin administration before injection of FDG. A few approaches to insulin administration have been proposed, but major criticisms include inappropriate insulin dose leading to either persistent hyperglycaemia or significant (9.5%) risk of hypoglycaemia [15], reduced imaging quality due to diffuse skeletal muscle and myocardial uptake due to glucose transporter type 4 (GLUT4)-mediated uptake in these tissues [16]. The latter effect has been reported to result in non-diagnostic images requiring a repeat study in approximately 25% of cases in one series [15]. In addition to the problem of rescheduling patients with non-diagnostic scans [17], the impact on workflow and patient inconvenience has limited the widespread adoption of this approach.

Since 2006, our centre developed an empiric protocol for intravenous insulin administration comprising approximately 10 IU Actrapid® (neutral insulin, Novo Nordisk) for BGL > 10 mmol/L, with variable adjustment for insulin sensitivity based upon extremes of body mass index (BMI) and—if applicable—the patient’s usual insulin requirements. The BGL is closely monitored following insulin injection until a nadir is reached (i.e., BGL clearly rising again), indicating resolution of biologic insulin effect. Although developed over a decade’s experience, limitations of this experiential approach include operator-dependence and lack of generalizability, in addition to potential delays deliberating over the choice of insulin dosage. We subsequently developed an insulin nomogram based upon the principle of the “correction factor” and “insulin sensitivity factor” utilised in personalised diabetic insulin pump therapy (see Additional file 1). This personalised insulin calculator (PIC) was introduced in 2015 to calculate an individualised insulin dosage based upon the degree of blood glucose correction required (determined by BGL at presentation) and estimated insulin sensitivity (determined from BMI and overall body weight). The aim of this audit is to establish the real-world safety, efficacy, and departmental workflow impact of this PIC in comparison to our previous practice.

## Materials and methods

### Patient selection and preparation

This study involved a retrospective review of all patients treated with insulin for hyperglycaemia (defined as BGL > 10 mmol/L) prior to FDG PET/CT at Peter MacCallum Cancer Centre over a 2-year period. The local institutional review board approved this study and the requirement to obtain informed consent was waived. Cohort 1 comprised a 12-month period using the department’s established empiric-dose insulin protocol (April 1, 2014–March 31, 2015), and Cohort 2 comprised the 12 months immediately following introduction of a personalised insulin calculator (April 1, 2015–March 31, 2016). There were 12,415 FDG PET/CT studies performed during the 2-year period. Keyword search of the departmental database Karisma© (Kestral, Melbourne, Australia) by patient and request conditions (using the terms “diabetic”, “IDDM” [insulin dependent diabetes mellitus] and “NIDDM” [non-insulin dependent diabetes mellitus), and report word search (using the terms “actrapid” and “insulin”) identified 351 patients who presented for FDG PET/CT scan with hyperglycaemia (including 14 with glucocorticoid-induced hyperglycaemia). Variables including age, gender, weight, height, body mass index (BMI), diabetes type, decision to administer insulin, insulin-dose calculated (Cohort 2) and/or administered, BGL at baseline and nadir, hypoglycaemia (defined as BGL < 3.5 mmol/L) and time to injection of FDG were analysed.

Patients were fasted for a minimum 6 h prior to presentation and—if known diabetic status—scheduled for early morning appointments to enable time for insulin administration. Short-acting insulin and oral hypoglycaemic agents were withheld the morning of the FDG PET/CT study.

### Cohort 1

The existing protocol for insulin prescription was as follows: insulin-naïve patients received between approximately 6–12 IU intravenous (IV) Actrapid® (neutral insulin, Novo Nordisk); those already treated with insulin were prescribed half the patient’s usual morning rapid-acting insulin dose as IV Actrapid. There was additional variation in insulin prescription depending upon extent of hyperglycaemia and individual nuclear medicine physician preference.

### Cohort 2

Patient’s BGL, weight (kg), and height (cm) was entered into the electronic PIC, and the calculated amount was prescribed as IV Actrapid® (per protocol [PP] group). The supervising nuclear medicine physician reserved the right to adjust the insulin dose according to preference, and a subset of patients were defined as the modified dose (MD) group if the prescribed insulin dose differed from the PIC recommendation by ± 1.0 IU. This occurred early in the study period, until the medical staff were confident in the safety and efficacy of the PIC. The outcomes of the entire Cohort 2 were also analysed (intention-to-treat [ITT] group).

### BGL monitoring and FDG administration

**Fig. 1:**
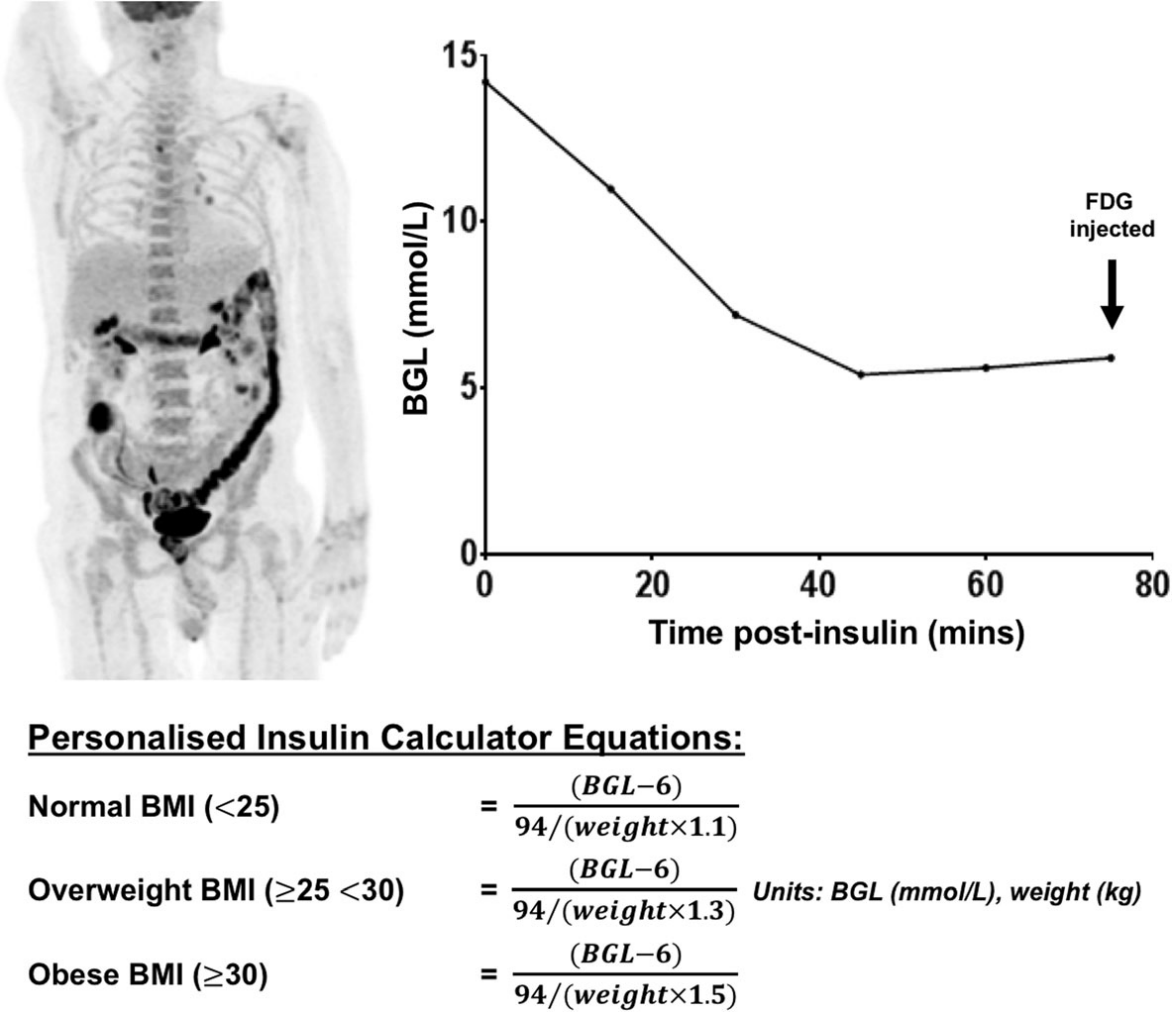
Case example demonstrating practical use of the insulin calculator. Seventy-three-year old man referred for FDG PET/CT staging of melanoma. Baseline BGL is 14.2 mmol/L (weight 75 kg and height 176 cm), and 7 IU Actrapid insulin was administered as per personalised insulin calculator recommendation. BGL declined to nadir of 5.4 mmol/L at 45 min, remaining stable prior to FDG injection. Maximum intensity projection shows diagnostic-quality FDG PET with appropriate target-to-background ratio and expected bio-distribution (no elevated skeletal muscle activity). Intense colon uptake associated with metformin administration noted. Personalised insulin calculator equations are provided (see Additional file 1 for details)

Venous BGL was monitored (Optium Xceed™ glucometer and Medisense Optium™ glucose test strips; Abbott Diabetes Care, CA, USA) at baseline and every 15 min after IV Actrapid® administration until the BGL had clearly reached a nadir and commenced rising again, indicating resolution of an insulin physiologic effect (minimum 60 min after Actrapid® administration). FDG was subsequently administered. If BGL remained > 10 mmol/L, a second dose of IV Actrapid® could subsequently be administered at the discretion of the supervising nuclear medicine physician with the same monitoring and FDG timing protocol applied. A typical case example using the PIC is described in Fig. 1.

### Data analysis

Descriptive statistics were reported as mean ± standard deviation (SD), and statistical analysis using a parametric unpaired *t*-test was performed to compare data from the two cohorts, unless otherwise specified. Mann-Whitney test was performed for comparison of non-parametric variables. Statistical analysis was performed using GraphPad Prism v7.02 (GraphPad Software, CA, USA). A *P* value of less than 0.05 was considered statistically significant.

## Results

**Fig. 2:**
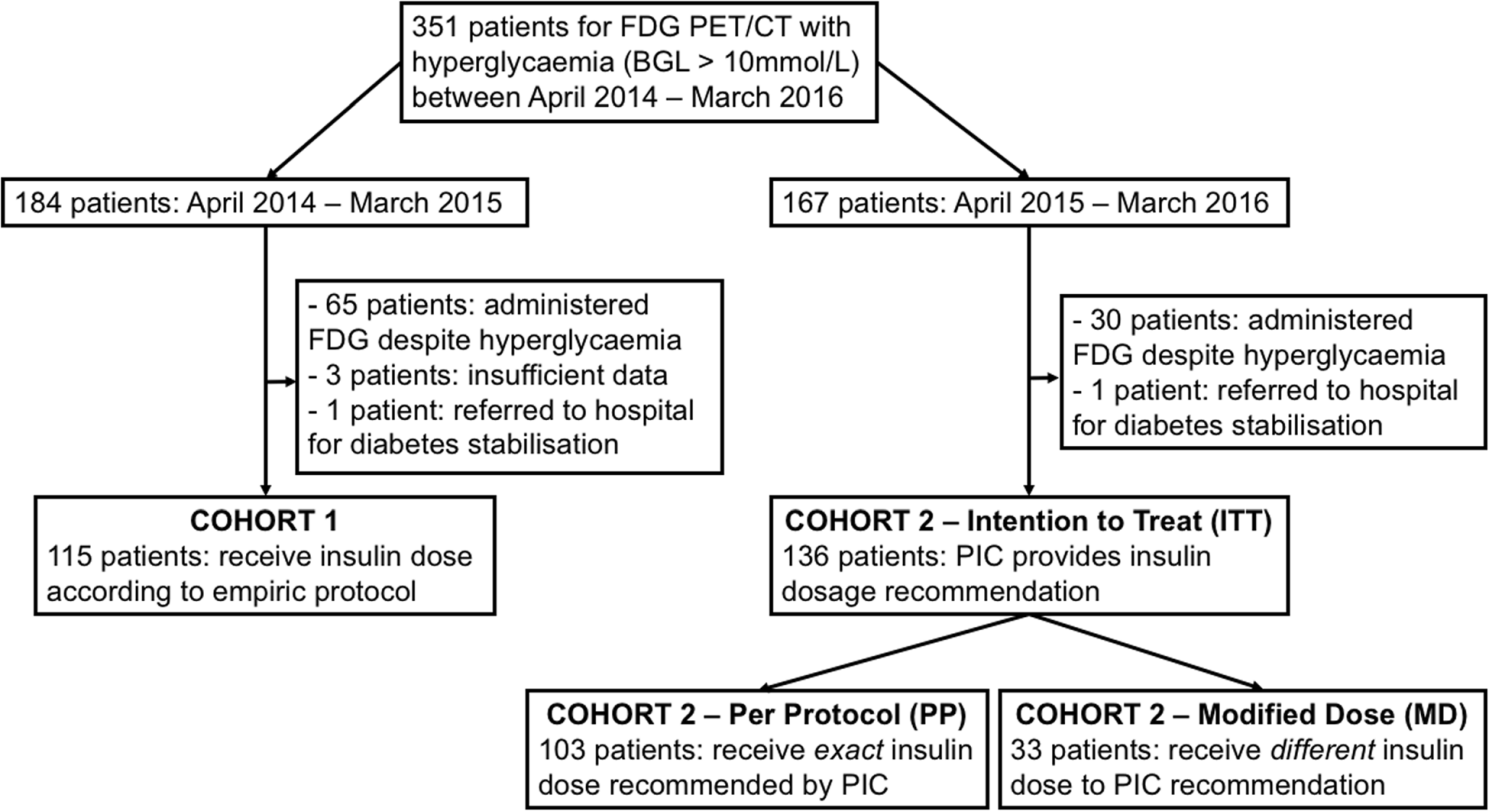
Consort diagram defining the study population. PIC, personalised insulin calculator; BGL, blood glucose level; ITT, intention-to-treat group; PP, per protocol group; MD, modified dose group

Three hundred fifty-one patients (242 males and 109 females) presented for FDG PET/CT with hyperglycaemia (BGL > 10 mmol/L). Definition of the study population is graphically represented in a consort diagram (Fig. 2). There were 184 patients in Cohort 1, of whom 65 (35.3%) patients proceeded with FDG PET/CT despite uncorrected hyperglycaemia (mean BGL 10.9 mmol/L ± 1.17), 3 patients had insufficient data recorded, 1 patient presenting with severe hyperglycaemia (31.2 mmol/L) received insulin in our department prior to referral for acute medical assessment and the remaining 115/184 (63.0%) patients (age 46–85 years) received insulin according to the empiric protocol. Of 167 patients in Cohort 2, 30 (18.0%) patients proceeded with FDG PET/CT despite uncorrected hyperglycaemia (mean BGL 11.2 mmol/L ± 1.28) and 1 patient that presented with severe hyperglycaemia (20.4 mmol/L) was referred for diabetes stabilisation and rescheduled. Of the remaining 136 insulin-treated patients (ITT group, age 43–92 years), 33 patients received an adjusted insulin dose that was different to that recommended by the PIC at the discretion of the treating nuclear medicine physician (MD group), leaving 103 patients who received the exact insulin dose according to the calculator (PP group). For the MD group, the mean difference (± standard deviation) between the insulin dosage recommended by PIC and prescribed by a nuclear medicine specialist was + 2.9 IU (± 1.9 IU). All patients treated according to the study protocol successfully completed FDG PET/CT of diagnostic quality on the day of scheduled appointment. No scans needed to be repeated due to altered bio-distribution following insulin administration.

There was no significant difference in baseline characteristics between Cohort 1 and Cohort 2 (Table 1). These groups had similar mean age (67 vs 66 years), gender (71% vs 70% male), weight (86.5 kg vs 89.5 kg), BMI (30.0 vs 30.7), known type 1 diabetes (7.8% vs 7.4%), type 2 diabetes (85.2% vs 88.2%), not known to be diabetic (7% vs 4.4%) and use of insulin (52.2% vs 46.3%) or oral diabetic therapies (75.6% vs 79.4%). The baseline BGL (14.5 vs 14.4 mmol/L) and range of BGL at presentation were also similar (10.5, 22.7 mmol/L) for Cohort 1 vs (10.4, 24.3 mmol/L) for Cohort 2 ITT.

**Table 1:** Baseline characteristics comparison between Cohort 1 and Cohort 2 (overall ITT group)

Characteristic	Cohort 1 *N = 115*	Cohort 2 (ITT) *N = 136*	*P* value
Age, (years) mean ± SD	67 ± 7.6	66 ± 8.5	NS
Male gender, *N* (%)	82 (71%)	95 (70%)	NS
Weight, (kg) mean ± SD	86.5 ± 18.6	89.5 ± 22.0	NS
BMI, (kg/m^2^) mean ± SD	30.0 ± 6	30.7 ± 6.6	NS
Diabetes type, *N* (%)			
- Type 1	9 (7.8%)	10 (7.4%)	NS
- Type 2	98 (85.2%)	120 (88.2%)	NS
- Unknown	8 (7.0%)	6 (4.4%)	NS
Diabetes therapy, *N (%)*			
- Insulin	60 (52.2%)	63 (46.3%)	NS
- Oral therapy	87 (75.6%)	108 (79.4%)	NS
BGL, (mmol/L)			
- Mean ± SD	14.5 ± 2.6	14.4 ± 2.6	NS
- Range	10.5–22.7	10.4–24.3	

*ITT* intention to treat, *SD* standard deviation, *N* number, *BMI* body mass index, *BGL* blood glucose level, *NS* not significant (*P* > 0.05)

Baseline characteristics between the PP and MD subgroups of Cohort 2 were also similar (Table 2), with the exception of existing insulin therapy for diabetes (37.9% [PP] vs 69.7% [MD], *P* < 0.0017) and weight (87 kg ± 20.8 [PP] vs 97 ± 23.9 kg [MD], *P* < 0.035) indicating that knowledge of patient’s existing insulin therapy and increased weight may have prompted the nuclear medicine physician to adjust the PIC recommended dosage.

**Table 2:** Baseline characteristics comparison within Cohort 2 (PP vs MD groups)

Characteristic	Cohort 2 (PP) *N = 103*	Cohort 2 (MD) *N = 33*	*P* value
Age, (years) mean ± SD	66 ± 9.1	65 ± 6.2	NS
Male gender, *N* (%)	69 (67%)	26 (79%)	NS
Weight, (kg) mean ± SD	87 ± 20.8	97 ± 23.9	< 0.035
BMI, (kg/m^2^) mean ± SD	30.3 ± 6.2	32 ± 7.6	NS
Diabetes type, *N* (%)			
- Type 1	8 (7.8%)	2 (6.1%)	NS
- Type 2	90 (87.4%)	30 (90.9%)	NS
- Unknown	5 (4.9%)	1 (3%)	NS
Diabetes therapy, *N (%)*			
- Insulin	39 (37.9%)	23 (69.7%)	< 0.002
- Oral therapy	57 (55.3%)	9 (27.3%)	NS
BGL, (mmol/L)			
- Mean ± SD	14.2 ± 2.7	14.8 ± 2.3	NS
- Range	10.4–24.3	10.4–19.5	

*PP* per protocol, *MD* modified dose, *SD* standard deviation, *N* number, *BMI* body mass index, *BGL* blood glucose level, *NS* not significant (*P* > 0.05)

Results of the analysis comparing the empiric insulin protocol (Cohort 1) vs the PIC (Cohort 2) as an overall ITT group and per protocol (PP) sub-group are summarised in Table 3.

**Table 3:** Comparative results following insulin administration patients in Cohort 1, Cohort 2 (ITT) and Cohort 2 (PP) groups

Outcome	Cohort 1 *N = 115*	Cohort 2 (ITT) *N = 136*	*P* value*	Cohort 2 (PP) *N = 103*	*P* value#
Hypoglycaemia (BGL < 3.5 mmol/L)	6 (5.2%)	1 (0.7%)	< 0.03	0 (0%)	< 0.019
Mean BGL at time of FDG, mmol/L	9.0 ± 2.6	8.6 ± 2.3	NS	8.6 ± 2.2	NS
Insulin dose prescribed, IU	12 ± 4.1	11.1 ± 5.1	NS	10.5 ± 4.9	NS
Mean BGL decline, mmol/L	7.1 ± 2.8	6.8 ± 2.9	NS	6.8 ± 2.9	NS
Achieved BGL ≤ 10.0 mmol/L, *N* (%)	92 (80%)	116 (85.3%)	0.27	91 (88.3%)	0.095
Repeat insulin dosage, *N* (%)	17 (14.8%)	10 (7.4%)	0.06	7 (6.8%)	0.06
Time from insulin to FDG injection, min					
- Median	136	108	< 0.001	108	< 0.001
- Range	68–287	70–237		75–237	

*ITT* intention to treat, *PP* per protocol, *N* number, *BGL* blood glucose level, *IU* international units of human insulin, *NS* not significant (*P* > 0.05); *unpaired *t*-test between Cohort 1 and Cohort 2 ITT group, #unpaired *t*-test between Cohort 1 and Cohort 2 PP group

### Hypoglycaemia (BGL < 3.5 mmol/L)

Overall, Cohort 2 (ITT) had significantly fewer hypoglycaemic events than Cohort 1 (1/136, 0.7% vs 6/115, 5.2%, *P* < 0.03). Notably, there were no hypoglycaemic events in the patients treated according to the PIC (Cohort 2 PP group), with the solitary case of hypoglycaemia within Cohort 2 occurring in the Cohort 2 MD group (BGL 13.8, PIC advised 5 IU Actrapid®, but 7 IU Actrapid® prescribed by a nuclear medicine physician).

Patients who developed hypoglycaemia had significantly lower mean weight (64.6 kg [hypoglycaemia] vs 88.2 kg [all patients], *P* = 0.0038) and BMI (23.1 [hypoglycaemia] vs 30.4 [all patients], *P* = 0.0005) than the remainder of the study participants. There was also a trend toward a lower baseline BGL (12.7 vs 14.4 mmol/L, *P* = 0.057) amongst patients with hypoglycaemia.

The majority of hypoglycaemic events were mild, asymptomatic and managed with close observation. One patient was symptomatic, treated with intravenous glucose solution and observed for approximately 3 h prior to FDG injection with subsequent acquisition of diagnostic-quality scan images. There were no incidents of severe hypoglycaemia, defined as hypoglycaemia requiring assistance due to impaired conscious state. All other patients experienced asymptomatic hypoglycaemia, received ongoing 15-minutely BGL and nursing observations and subsequently received FDG injection without incident.

### Hyperglycaemia (BGL > 10 mmol/L)

Overall, both protocols were effective, with similar mean BGL at time of FDG administration between these groups (Cohort 1 9.0 ± 2.6 mmol/L vs Cohort 2 ITT 8.6 ± 2.3 mmol/L vs Cohort 2 PP 8.6 ± 2.2 mmol/L). However, more patients in Cohort 1, 23/115 (20%), failed to achieve a target BGL < 10.0 mmol/L compared with the ITT group, 20/136 (14.7%), or PP group, 12/103 (11.7%). There was also a trend toward a greater number of patients requiring a repeat dose of insulin in Cohort 1, 17/115 (14.8%), compared with Cohort 2, ITT 10/136 (7.4%, *P* = 0.059) or PP sub-group 7/103 (6. 8%, *P* = 0.06), although this did not reach statistical significance.

### Per protocol vs modified dose groups

Overall, there was a similar mean administered insulin dose in the PP vs MD groups (10.5 vs 13 units). Notably, the only hypoglycaemic event of Cohort 2 occurred in the MD group, suggesting a greater risk of hypoglycaemia in the cases where the clinician adjusted the dose estimated by the PIC. Specifically analysing the 33 patients within the MD group, 17 patients received a higher and 16 patients received a lower dose than recommended by PIC. The mean (and maximum) dose increase and decrease was + 3.3 units (+ 9 units) and − 2.3 units (− 5.5 units), respectively.

### Logistical considerations

There was a significant difference in median time between Actrapid injection and FDG injection between Cohort 1 (136 min [68–287]) and Cohort 2 (ITT 108 min [70–237], *P* < 0.001 and PP 108 min [75–237], *P* < 0.001), using the Mann-Whitney test. There was a greater individualised range of insulin prescription using the PIC in Cohort 2 ITT, 3–32 units, compared to Cohort 1, 4–20 units. Importantly, all scans in this series were of diagnostic quality without need for any scans to be repeated.

## Discussion

Our findings confirm that the personalised insulin calculator is safe and effective for the correction of hyperglycaemia prior to FDG administration. It demonstrated significantly fewer hypoglycaemic events than the previous empiric protocol by both ITT and PP analysis (0.7% ITT and 0% PP versus 5.2% empiric protocol). Notably, the only hypoglycaemic event in Cohort 2 ITT population occurred due to nuclear medicine specialist decision to increase the prescribed insulin dosage above that recommended by the calculator. Consequently, increase of insulin above the dose recommended by the PIC is not advisable. Importantly, there was a significantly shorter time interval using the PIC between insulin and FDG injections (108 vs 136 min), representing fewer repeated insulin injections. Although not assessed in our retrospective study, anecdotally, the PIC is also faster and simpler than a nuclear medicine specialist’s determination of insulin dose and is a major advantage of this approach. The PIC is also more effective, with more patients achieving a target BGL of 3.5–10.0 mmol/L and fewer repeat insulin injections required to achieve this.

Despite numerous papers and guidelines (SNM, EANM, NCI) proposing insulin regimens for correction of hyperglycaemia, few provide supporting data on the safety and efficacy of their approach to achieve this. Roy et al. recommend initial administration of intravenous short-acting Humulin-R insulin at a significantly lower dosage (2 units for glycaemia of 10.0–12.0, 3 units for glycaemia of 12.1–14.0, and 4–6 units for glycaemia of 14.1 mmol/L and above, and the opportunity to administer additional insulin injections if the BGL persisted > 10.0 mmol/L at 30 min) [15]. By comparison, the mean recommended insulin dosage by the PIC in Cohort 2 (11.1 units) was approximately twice the highest recommended dosage of this sliding scale. Consequently, the number of injections required, cumulative insulin dose per patient and thus elapsed time of this sliding scale approach to insulin administration was likely to be significantly greater than our personalised approach. This approach also resulted in unacceptably high hypoglycaemia rates of 9.5% (compared with 0% in our PP analysis), presumably due to a cumulative effect of administered insulin. Otherwise, mean baseline BGL and BGL at time of FDG injection was similar in the Roy et al. study compared to Cohort 2 (13 ± 2 mmol/L vs 14.6 ± 2.3 mmol/L and 7 ± 2 mmol/L vs 8.6 ± 2.3 mmol/L). Caobelli et al. used an intravenous insulin infusion which effectively achieved euglycaemia in all 20 cases, without hypoglycaemia. However, 5% of cases had inadequate scan bio-distribution and greater nursing input was required [18].

Other groups also suggested sliding scale regimens but did not provide any evidence to support the safety or efficacy of their recommendations. Surasi et al. advised a sliding scale regimen (2 units for glycaemia of 11.1–13.9; 3 units for glycaemia of 14.0–16.7; 4 units for glycaemia of 16.8–19.4; and 5 units for glycaemia of 19.5–22.2), but with lower recommended insulin dosages. IAEA suggest 1–2 units may be used for BGL > 11.1 mmol/L. [19]. The SNM procedure guideline suggests that insulin may be used to lower BGL but without specific advice on dosage or delay prior to FDG administration [11]. The EANM guidelines state that the preferred insulin administration is rapid acting insulin via subcutaneous injections, requiring an inconvenient 4-h delay prior to FDG injection [13].

Personalised insulin calculation is a key advantage over previously proposed insulin protocols that depend upon a generic sliding scale regimen. Whilst most prior insulin recommendations recognised that a higher baseline BGL requires a higher insulin dose, there was no allowance for variation in insulin sensitivity between patients. This was a major criticism of prior protocols, because “the extent of glycaemic reduction is not predictable and the chance of study failure is unavoidable [17].” In addition to intrinsic inaccuracy, this would also limit applicability between patient populations with different rates of insulin sensitivity. Our previous empiric protocol (with a prescribed insulin range of 4–20 varied by numerous factors including BMI, BGL and usual insulin dosage) considered this, but was dependent upon the department’s cumulative experience rather than a defined protocol readily applicable to other sites or staff inexperienced in this technique. In comparison, the personalised insulin calculator estimates the insulin sensitivity of each patient based upon BMI and total body weight, considers the magnitude of required BGL reduction and is likely to be readily transportable to sites with different patient populations and independent of clinician experience.

This novel personalised approach also applies to the subsequent individualised timing of FDG injection. In addition to minimising the risk of unrecognised hypoglycaemia, regular 15-minutely BGL monitoring after insulin administration until it reaches a nadir enables identification of the resolution of physiologic insulin effect. This approach minimises the time after insulin injection whilst maximising departmental efficiency and avoiding problems associated with premature insulin administration (especially increased muscle FDG uptake due to ongoing insulin action via GLUT4-mediated-facilitated glucose uptake). An elegant early study by Lindholm et al. neatly demonstrated a “sink effect” of increased muscle uptake and markedly poor scan quality due to endogenous hyperinsulinaemia 1 h after glucose loading as the explanation for a significant (~ 50%) reduction in SUVmax in head and neck malignancy, compared to studies performed in the fasting state [8]. Roy et al. observed a significantly increased risk of non-diagnostic scans at shorter intervals after insulin injection (minimum 60-min post-iv insulin), and also patients with a greater insulin-induced fall in BGL when compared to patients with diagnostic-quality scans. This is further supported by Garcia et al. who demonstrated 60% suboptimal PET/CT studies in patients that were injected with FDG whilst the BGL level was falling after subcutaneous insulin injection (30–115 min); all patients injected with FDG after 4 h had optimal studies [20]. Although a standardised delay of 90-min post-iv, or 240-min after subcutaneous insulin injection [13] may be effective, some patients could be safely administered FDG earlier, and others may potentially require a longer delay. Our department routinely monitors BGL 15-minutely after insulin injection to identify the nadir at which point the BGL starts to rise, indicating wearing-off of peripheral insulin action and ability to inject FDG without unwanted skeletal muscle uptake. The intrinsic variability of glucose monitors must be considered, and if there is doubt that the BGL has started to rise, a confirmatory repeat measurement 15 min later is recommended. In our patients, FDG was safely administered at the earliest ~ 70 min (noting that some patients required significantly longer delays), and no patients required repeat imaging for non-diagnostic scans. In contrast, other groups have recommended a minimum 60-min delay between insulin and FDG injection, potentially explaining observed muscle uptake. Patients with diabetes are advised to present approximately 90 min prior to their scheduled scan to minimise disruption to workflow by allowing time for insulin administration if necessary.

The personalised insulin calculator demonstrated a marked reduction in hypoglycaemia, with no episodes occurring in patients administered insulin according to the calculator’s recommendations (ITT group). The single episode in Cohort 2 occurred in a patient prescribed a higher than recommended dose of insulin (MD group). In contrast, hypoglycaemia occurred in 5.2% of cases in our control group (Cohort 1) and 9.5% of patients in a previous study [15]. There were no episodes of severe hypoglycaemia with either protocol in our study. The comparatively favourable hypoglycaemia rate in our control group emphasises the quality of the existing insulin protocol prior to implementation of this nomogram. It is anticipated that the impact of the PIC (e.g. time to FDG injection, need for repeat injections, incidence of hypoglycaemia) may be greater at other departments with less experience in pre-FDG insulin administration. Patients in our study who experienced hypoglycaemia had significantly lower weight and BMI and a trend toward a lower baseline BGL. These variables would be associated with a lower insulin requirement due to increased insulin sensitivity and reduced need for BGL reduction. Although not specifically examined in our study, other groups known to be at higher risk of hypoglycaemia include type 1 diabetes mellitus (reduced pancreatic endocrine counter-regulatory response), renal impairment (delayed insulin clearance) and during withdrawal of glucocorticoid therapy (reduced adrenal endocrine counter-regulatory response) [21].

The details of our personalised insulin calculator are described in the Additional file 1 and a web-based calculator is freely available at https://www.petermac.org/services/diagnosis-investigations/positron-emission-tomography-pet/fdg-pet-insulin-calculator. Whilst insulin was safely administered to patients with BGL up to 24.3 mmol/L, we emphasise that the personalised insulin calculator is a tool to guide selection of insulin to correct adult hyperglycaemia but the clinical decision to administer insulin to a particular patient remains the responsibility of the prescribing clinician. Whilst insulin administration is safe in patients with acute hyperglycaemia, there is a risk of hypokalaemia and cerebral oedema associated with rapid correction of chronic hyperglycaemia in patients with hyperosmolar hyperglycaemic state [22] and clinical assessment for evidence of dehydration and/or impaired conscious state is recommended. We would routinely consider rescheduling patients with BGL > 18 mmol/L and referring for diabetic optimisation.

It is important to emphasise that optimal management of hyperglycaemia in preparation for FDG PET/CT requires a multifaceted approach. Generally, this includes scheduling diabetic patients in the early morning after an overnight fast to ensure adequate time for correction of hyperglycaemia if necessary, and proactive optimisation of diabetic control in days prior to the scan [17]. Proactive optimisation of diabetic control is preferable if possible but is resource intensive requiring either frequent communication with a nuclear medicine physician or endocrinologist in the days leading up to the scan [23]. Given the routine clinical challenges optimising diabetic control in an outpatient setting, this approach is ambitious and at risk of failure in a “real-world” setting, particularly given pressure for scheduling PET scans in a busy department prior to important deadlines including multidisciplinary team meetings and medical appointments. This is especially important for patients who need to travel a significant distance to access imaging services. Implementation of this protocol may require adjustment to departmental staffing and workflow, including doctor availability to prescribe insulin and nursing staff for insulin administration and BGL monitoring.

There are several limitations of this retrospective study. Data pertaining to renal function and type 1 diabetes diagnosis was not available for all patients, limiting firm conclusions relating to the risk of hypoglycaemia in these patient sub-groups. It is also possible that some patients may not have been identified if details of insulin administration were omitted in the report. However, this is estimated to be a small number, and unlikely to bias the results. Furthermore, administration of insulin was at the discretion of the physician, and in a minority of cases, the nuclear medicine specialist modified the recommended dose of insulin. However, it is noted that more patients experienced hypoglycaemia and uncorrected hypoglycaemia in this cohort suggesting worse outcomes when the nuclear medicine specialist modifies the recommended insulin dose. Notably, the proportion of hyperglycaemic patients undergoing FDG PET/CT without insulin administration significantly reduced with the introduction of the calculator, suggesting increased confidence with this approach.

Finally, although the insulin calculator considers the patient’s BMI and total body weight, other factors contribute to insulin sensitivity (including ethnicity, age, gender, physical activity and renal function) and may theoretically differ between populations. However, the PIC was developed in a city recognised for its diverse multicultural population (Melbourne, Australia) and the absence of significant hypoglycaemia when used in this varied population is reassuring. The PIC was tested in a population with age ranging from 43 to 92 years and use in patients outside of this age range must be done with caution. In particular, no paediatric patients were included in this cohort and PIC cannot be recommended in this population without further study.

## Conclusion

Our personalised insulin calculator is safe and effective for the correction of hyperglycaemia prior to FDG PET/CT, with markedly fewer hypoglycaemic events and significantly reduced time between insulin and FDG administration compared to an empiric protocol. All scans obtained were of diagnostic quality. This web-based calculator is freely available via a website (https://www.petermac.org/services/diagnosis-investigations/positron-emission-tomography-pet/fdg-pet-insulin-calculator) and could be easily adopted at other sites.

## Additional file

Additional file 1: Background to the development of personalised insulin calculator equations. (DOCX 15 kb)

## Abbreviations

BGL: Blood glucose level
EANM: European Association of Nuclear Medicine
FDG: ^18^F-fluorodeoxyglucose
GLUT4: Glucose transporter type 4
IAEA: International Atomic Energy Agency
IDDM: Insulin-dependent diabetes mellitus
ITT: Intention-to-treat
IU: International units
MD: Modified dose
NCI: National Cancer Institute
NIDDM: Non-insulin-dependent diabetes mellitus
PET/CT: Positron emission tomography/computed tomography
PIC: Personalised insulin calculator
PP: Per protocol
SD: Standard deviation
SNM: Society of Nuclear Medicine
SUVmax: Maximum standardised uptake value
