# Supplementary material for: Personalised insulin calculator enables safe and effective correction of hyperglycaemia prior to FDG PET/CT
Source: EJNMMI Res. 2019 Feb 8;9:15. doi: 10.1186/s13550-019-0480-2 (PMC6368634; doi:10.1186/s13550-019-0480-2)
Supplement: Supplementary file 1 — Background to the development of personalised insulin calculator equations. (DOCX 15 kb) [file 13550_2019_480_MOESM1_ESM.docx]

**Additional file 1**

The personalised insulin calculator was developed according to the principle of the insulin correction factor used for treating hyperglycaemia with insulin pump therapy. This considers the difference between the patient’s baseline and target BGL and utilises a personalised ‘insulin sensitivity factor’ (ISF), which is defined as the drop in blood glucose level, caused by each unit of administered insulin. Consequently, the amount of insulin required to correct hyperglycaemia back to ‘target BGL’ is estimated as follows:

Amount of insulin required = (BGL – target BGL) / Insulin sensitivity factor

There are various methods to calculate the insulin sensitivity factor, and the method followed is defined as the “1700 rule”, involving 1700 divided by the total daily dose (TDD) of insulin^1^. Other published figures vary between 1400 – 1960, depending on patient population studied and use of short or rapid-acting insulin^2^.

Insulin sensitivity factor = 1700 / TDD mg/dL/unit

= 94 / TDD mmol/L/unit

The insulin TDD can be estimated from the patient’s body weight (typically 0.6 units/kg^3^), but higher and more variable requirements are necessary for patients with type 2 diabetes mellitus due to varied insulin sensitivity.

This initial algorithm was subsequently validated against data collected from a reference population of 170 patients previously treated with insulin to correct hyperglycaemia prior to FDG PET/CT at Peter MacCallum Cancer Centre between November 2006 and July 2012. This retrospective analysis identified two subgroups of patients whom had an unsatisfactory response to insulin administration, defined as low BGL nadir (BGL < 4.0 mmol/L) or high BGL nadir (BGL > 10.0 mmol/L). An iterative process adjusted the target BSL and ISF variables in the insulin calculator, seeking to optimise the algorithm such that a lower insulin dose is recommended to cases in the ‘low BGL nadir’ group, and a higher insulin dose is recommended to those in the ‘high BGL nadir’ group. In addition to consideration of the patient’s weight, the correction factor ‘ISF’ was converted to mmol/L, and scaled depending upon the BMI class as a surrogate for insulin resistance (normal weight BMI < 25 kg/m2, overweight if BMI >= 25 but < 30kg/m2 and obese if BMI >= 30), following similar principles of the Endocrine Society guidelines and University of Pittsburgh Medical Centre protocol for management of hyperglycaemia in hospitalised patients in non-critical care setting4^,^5.

The final protocol appropriately recommended a lower insulin dose in 13/13 (100%) cases in the ‘low BGL nadir’ group, and a higher insulin dose in 25/28 (89.3%) cases in the ‘high BGL nadir’ group. Please refer to main text for explanation regarding safety, efficacy and practical guidance regarding use of this personalised insulin calculator, which is freely available at <https://www.petermac.org/services/diagnosis-investigations/positron-emission-tomography-pet/fdg-pet-insulin-calculator>.

Personalised Insulin Calculator:

Normal BMI (<25) = (BGL-6)/(94/(weight*1.1))

Overweight BMI (>=25 < 30) = (BGL-6)/(94/(weight*1.3))

Obese BMI (>=30) = (BGL-6)/(94/(weight*1.5))

*Units: BGL (mmol/L), weight (kg)*

**REFERENCES:**

Davidson PC, Hebblewhite HR, Bode BW, et al. Statistically based CSII parameters: correction factor (CF) (1700 rule), carbohydrate-insulin ratio (CIR) (2.8 rule), and basal-to-total ratio. *Diabetes Technol Ther*. 2003; 5: 237

2 King AB, Kuroda A, Matsuhisa M, Hobbs T. A Review of Insulin-Dosing Formulas for Continuous Subcutaneous Insulin Infusion (CSII) for Adults with Type 1 Diabetes. *Curr Diab Rep*. 2016; 16: 83.

3 Rubin DJ, Rybin D, Doros G, McDonnell ME. Weight-based, insulin dose-related hypoglycaemia in hospitalized patients with diabetes. *Diabetes Care*. 2011; 34: 1723-1728.

4 Umpierrez GE, Hellman R, Korytkowski MT, et al. Management of Hyperglycaemia in Hospitalised patients in Non-Critical Care Setting: An Endocrine Society Clinical Practice Guideline. *J Clin Endocrinol Metab.* 2012; 97: 16-38.

5 Magaji V, Johnston JM. Inpatient Management of Hyperglycaemia and Diabetes, derived from protocol used by University of Pittsburgh Medical Centre. *Clinical Diabetes*. 2011; 29: 3-9.
